# Impact of proton pump inhibitor use on clinical outcomes in East Asian patients receiving clopidogrel following drug-eluting stent implantation

**DOI:** 10.1186/s12916-024-03549-y

**Published:** 2024-08-15

**Authors:** Ju Hyeon Kim, Soon Jun Hong, Jung-Joon Cha, Subin Lim, Hyung Joon Joo, Jae Hyoung Park, Cheol Woong Yu, Tae Hoon Ahn, Young-Hoon Jeong, Byeong-Keuk Kim, Kiyuk Chang, Yongwhi Park, Young Bin Song, Sung Gyun Ahn, Jung-Won Suh, Sang Yeub Lee, Jung Rae Cho, Ae-Young Her, Hyo-Soo Kim, Moo Hyun Kim, Eun-Seok Shin, Do-Sun Lim

**Affiliations:** 1 Department of Cardiology, Korea University Anam Hospital, Korea University College of Medicine, Cardiovascular Center 73, Goryeodae-Ro, Seongbuk-Gu, Seoul, 02841 Republic of Korea; 2 Department of Cardiology, Heart and Brain Institute, Chung-Ang University Gwang-Myeong Hospital, Chung-Ang University College of Medicine, Gwangmyeong-Si, Republic of Korea; 3 Severance Cardiovascular Hospital, Seoul, Republic of Korea; 4 Division of Cardiology, Department of Internal Medicine, College of Medicine, Catholic University of Korea, Seoul, Republic of Korea; 5 Department of Internal Medicine, School of Medicine and Cardiovascular Center, Gyeongsang National University, Gyeongsang National University Changwon Hospital, Changwon, Republic of Korea; 6 Division of Cardiology, Department of Medicine, Samsung Medical Center, Sungkyunkwan University School of Medicine, Seoul, Republic of Korea; 7 Department of Cardiology, Yonsei University Wonju Severance Christian Hospital, Wonju, Republic of Korea; 8 Department of Internal Medicine, Department of Cardiology, Seoul National University College of Medicineand Seoul National University Bundang Hospital, Seongnam, Republic of Korea; 9 Cardiology Division, Department of Internal Medicine, Kangnam Sacred Heart Hospital, Hallym University College of Medicine, Seoul, Republic of Korea; 10 Division of Cardiology, Department of Internal Medicine, Kangwon National University School of Medicine, Chuncheon, Republic of Korea; 11 Department of Internal Medicine and Cardiovascular Center, Seoul National University Hospital, Seoul, Republic of Korea; 12 Department of Cardiology, Dong-A University Hospital, Busan, Republic of Korea; 13 Division of Cardiology, Ulsan University Hospital, University of Ulsan College of Medicine, Ulsan, Republic of Korea

**Keywords:** Clopidogrel, Proton pump inhibitor, Platelet reactivity, Poor metabolizer, Myocardial infarction, Bleeding

## Abstract

**Background:**

Concomitant use of clopidogrel and proton pump inhibitor (PPI) is common, but PPI may reduce the antiplatelet effects of clopidogrel in patients undergoing percutaneous coronary intervention (PCI). We evaluated the impact of PPI use on clinical outcomes in post-PCI patients, by incorporating P2Y12 reaction unit (PRU) and *CYP2C19* genotyping results.

**Methods:**

From a multicenter registry of patients who underwent PCI with drug-eluting stent implantation and received clopidogrel-based dual antiplatelet therapy (DAPT), patients who were prescribed a PPI at the time of PCI (PPI users) were compared to those who were not (non-users). The primary outcome included all-cause death, myocardial infarction, stent thrombosis, or cerebrovascular accident at 12 months. Major bleeding (Bleeding Academic Research Consortium [BARC] types 3–5) and gastrointestinal (GI) bleeding (BARC types 3–5) were important secondary outcomes. The adjusted outcomes were compared using a 1:1 propensity-score (PS) matching and competing risk analysis.

**Results:**

Of 13,160 patients, 2,235 (17.0%) were prescribed PPI, with an average age of 65.4 years. PPI users had higher on-treatment PRU levels than non-users. After PS matching, the primary outcome occurred in 51 patients who were PPI users (cumulative incidence, 4.7%) and 41 patients who were non-users (cumulative incidence, 3.7%; log-rank *p* = 0.27). In carriers of both *CYP2C19* loss-of-function alleles, PPI use was linked to an increased risk of the primary outcome (hazard ratio, 3.22; 95% confidence interval, 1.18–8.78). The incidence of major bleeding and GI bleeding (BARC types 3–5) was comparable between PPI users and non-users in the PS-matched cohort.

**Conclusions:**

In post-PCI patients receiving clopidogrel-based DAPT, PPI use was not linked to an increased risk of adverse cardiac and cerebrovascular events, but there was a small but significant increase in on-treatment PRU. Future research using a more individualized approach would further elucidate these interactions and guide evidence-based clinical practices.

**Supplementary Information:**

The online version contains supplementary material available at 10.1186/s12916-024-03549-y.

## Background

Patients receiving dual antiplatelet therapy (DAPT) after percutaneous coronary intervention (PCI) with drug-eluting stent (DES) implantation are at risk of both ischemic and bleeding complications. Proton pump inhibitors (PPIs) are commonly prescribed in these patients to treat or prevent gastrointestinal (GI) bleeding [1, 2]. Clopidogrel is a prodrug requiring bioactivation by the liver enzyme CYP2C19 (cytochrome P450, family 2, subfamily C, polypeptide 19) [3], whereas PPIs are primarily metabolized by the same enzyme into inactive metabolites [4]. This competitive inhibition has raised concerns regarding clinically relevant drug-drug interactions that could diminish the effectiveness of clopidogrel [5, 6]. In addition, the presence of *CYP2C19* loss-of-function (LoF) alleles increases the likelihood of PPI-clopidogrel interaction [7, 8].

Some randomized clinical trials (RCTs) and observational studies have shown contradictory results regarding the impact of PPI coadministration on clopidogrel efficacy, with some studies indicating attenuated P2Y12 receptor inhibition and increased adverse clinical outcomes [8–12]. However, previous studies have predominantly focused on metabolic interactions assessed using platelet function test in a limited patient population [13–16]. The largest RCT (PPI users; *n* = 1,876) found no significant cardiovascular interaction between clopidogrel and omeprazole, but the study’s predominantly white population (94%) suggests an underestimation of homozygosity for the *CYP2C19* LoF allele [8]. No research has investigated drug-drug-gene interactions on platelet reactivity and clinical outcomes in patients receiving clopidogrel-based DAPT after DES implantation.

The East Asian population has a higher prevalence of *CYP2C19* LoF allele than that of the Western population [17, 18], but they are significantly underrepresented in landmark RCTs. East Asian patients exhibit a distinct clinical profile characterized by fewer thromboembolic events and increased bleeding risk during antithrombotic therapies [19]. Due to this East Asian paradox, clopidogrel remains the most commonly prescribed platelet inhibitor in clinical practice because of its lower bleeding profile [20–22]. To address these gaps, a large-scale, observational, multicenter Platelet function and genoType-Related long-term proGnosis in DES-treated patients (PTRG-DES) study in South Korea included regular platelet reactivity testing (before and after clopidogrel loading) and *CYP2C19* genotyping [23–25]. From the PTRG-DES study, we sought to evaluate the safety of the concomitant use of PPI and clopidogrel-based DAPT on ischemic and bleeding outcomes using propensity-score (PS) matching analysis.

## Methods

### Source of data and study population

The PTRG-DES is a nationwide registry supported by the Korean Society of Interventional Cardiology (NCT04734028). The detailed study design and complete inclusion and exclusion criteria have been previously described [23–25]. Between January 2003 and December 2018, 33 academic centers in South Korea enrolled 13,160 consecutive patients who underwent PCI with DES implantation and were treated with clopidogrel-based DAPT. Patients who received P2Y12 inhibitors other than clopidogrel and those who required oral anticoagulants were excluded from the study. The institutional review board of each participating center approved the PTRG-DES registry (Korea University Anam Hospital; 2018AN0283) and waived the requirement for written informed consent for access to an institutional registry.

### Study measurements and procedures

After ensuring that the antiplatelet effects would last for a long enough time, the VerifyNow assay (Accriva, San Diego, CA, USA) was performed to measure the P2Y12 reaction unit (PRU) during the peri-procedural time [26]. Aspirin was administered in either a 300 mg coated oral dosage at least 2 h before PCI or a 100 mg dose at least 5 days before PCI. Clopidogrel was administered in doses of 600 mg at least 6 h before PCI, 300 mg at least 12 h before PCI, or 75 mg at least 5 days before PCI. No patient receiving abciximab was enrolled because of the long washout period. If eptifibatide or tirofiban was used during PCI, a 24-h washout period was required before VerifyNow testing [23]. High platelet reactivity (HPR) was defined as an on-treatment PRU > 208 [27].

Pyrosequencing of each single nucleotide polymorphism was performed for genotyping using commercially available analyzers such as the PSQ 96MA Pyrosequencer (Pyrosequencing AB, Uppsala, Sweden), the ABI PRISM® 3100 genetic analyzer (Applied Biosystems, Foster City, CA, USA), or the Spartan RXTM system (Spartan Bioscience, Ottawa, Canada) [28–30]. The major Korean alleles include CYP2C19*2 (rs4244285), CYP2C19*3 (rs4986893), and CYP2C19*17 (rs12248560). Extensive metabolizers include CYP2C19*1/*1 and CYP2C19*1/*17. Intermediate metabolizers include CYP2C19*1/*2, CYP2C19*1/*3, CYP2C19*2/*17, and CYP2C19*3/*17. Poor metabolizers include CYP2C19*2/*2, CYP2C19*2/*3, and CYP2C19*3/*3. The physicians and patients were not informed of the PRU and *CYP2C19* genotyping results.

All PCI procedures were performed according to current guidelines. During PCI, parenteral anticoagulation was used to maintain an active clotting time of 250–300 s. The operator selected the treatment method, stent type, diameter, length, and drug use. The index PCI guidelines recommend DAPT with aspirin and clopidogrel maintenance doses for at least 1 year. The DAPT duration was at the discretion of the attending physician.

### Proton pump inhibitors and study outcomes

PPIs were prescribed at the discretion of the treating physician and documented as a drug class in the case report form at the time of the PCI. However, specific PPIs and dosages were not reported in detail. Anemia was defined as a hemoglobin level < 13 g/dL in men and < 12 g/dL in women. Chronic kidney disease (CKD) was defined as an estimated glomerular filtration rate < 60 mL/min/1.73 m^2^.

The primary outcome was the incidence of major adverse cardiac and cerebrovascular events (MACCE) including all-cause death, myocardial infarction (MI), stent thrombosis, or cerebrovascular accident at 12 months after the index PCI. All-cause mortality, major bleeding (Bleeding Academic Research Consortium [BARC] types 3–5), and GI bleeding (BARC types 3–5) were important secondary outcomes. MI was defined as an increase in creatine kinase-myoglobin binding above the upper normal limit or a troponin T/I level > 99th percentile of the upper normal limit accompanied by ischemic symptoms, electrocardiographic abnormalities, or abnormal imaging findings indicative of ischemia. Any new embolic, thrombotic, or hemorrhagic stroke with neurologic impairments lasting for at least 24 h was considered a cerebrovascular accident. Unless an undeniable non-cardiovascular cause was discovered, all deaths were categorized as cardiovascular deaths. Patient interviews and medical records were used to obtain the demographic, angiographic, and procedural data. All follow-up visits were conducted in the form of office visits or telephone calls, where necessary. An independent committee blinded to the genetics and PRU data examined and adjudicated all clinical events from each participating site.

### Statistical analysis

Categorical variables are reported as counts and percentages, whereas continuous variables are presented as means and standard deviations. Group comparisons were performed using a parametric unpaired t-test or non-parametric Mann–Whitney U test for continuous variables and χ^2^ or Fisher’s exact test for categorical variables. To reduce the effect of selection bias, we conducted PS matching analysis to compare the adverse clinical events between PPI users and non-users. Using multivariable logistic regression, we assessed the likelihood of receiving PPIs during the index hospitalization. The variables were presentation as acute MI, age, sex, obesity, dyslipidemia, smoking, CKD, anemia, congestive heart failure, peripheral arterial disease, previous MI, previous coronary artery bypass graft, previous PCI, hemoglobin level, platelet count, glomerular filtration rate, baseline PRU, multivessel disease, bifurcation lesion, chronic total occlusion, PCI of the left main or left anterior descending artery, discontinuation of DAPT within 1 year, and the use of renin-angiotensin system inhibitor, statin, calcium channel blocker, and aspirin. We matched each patient in PPI users with those in non-users at a 1:1 ratio using the optimal method, with a caliper width equal to 0.2 of the standard deviation of the logit PS. The balance of baseline features was examined, and a standardized mean difference < 0.1 indicated a negligible difference.

During the follow-up period, patients were censored at the time of the event or date of their last follow-up. Only the first event was counted in patients with multiple events reported for the same outcome. Cumulative incidence rates were calculated based on Kaplan–Meier estimates, and intergroup comparisons were assessed using the log-rank test. A multivariable Cox proportional hazard regression model was used to analyze the influence of different covariates on time-to-event outcomes by calculating hazard ratios (HRs) and 95% confidence intervals. The sub-distribution HR for the primary outcome was estimated using the Fine-Gray competing risk model [31], and the discontinuation of DAPT was modelled as a single competing event. Subgroup analyses were performed according to the presence of hypertension, diabetes mellitus, *CYP2C19* genotyping, and HPR. Statistical analyses were performed using R version 4.1.2 (R Foundation for Statistical Computing, Vienna, Austria) with a value of *p* < 0.05 considered statistically significant.

## Results

### Study population

**Fig. 1:**
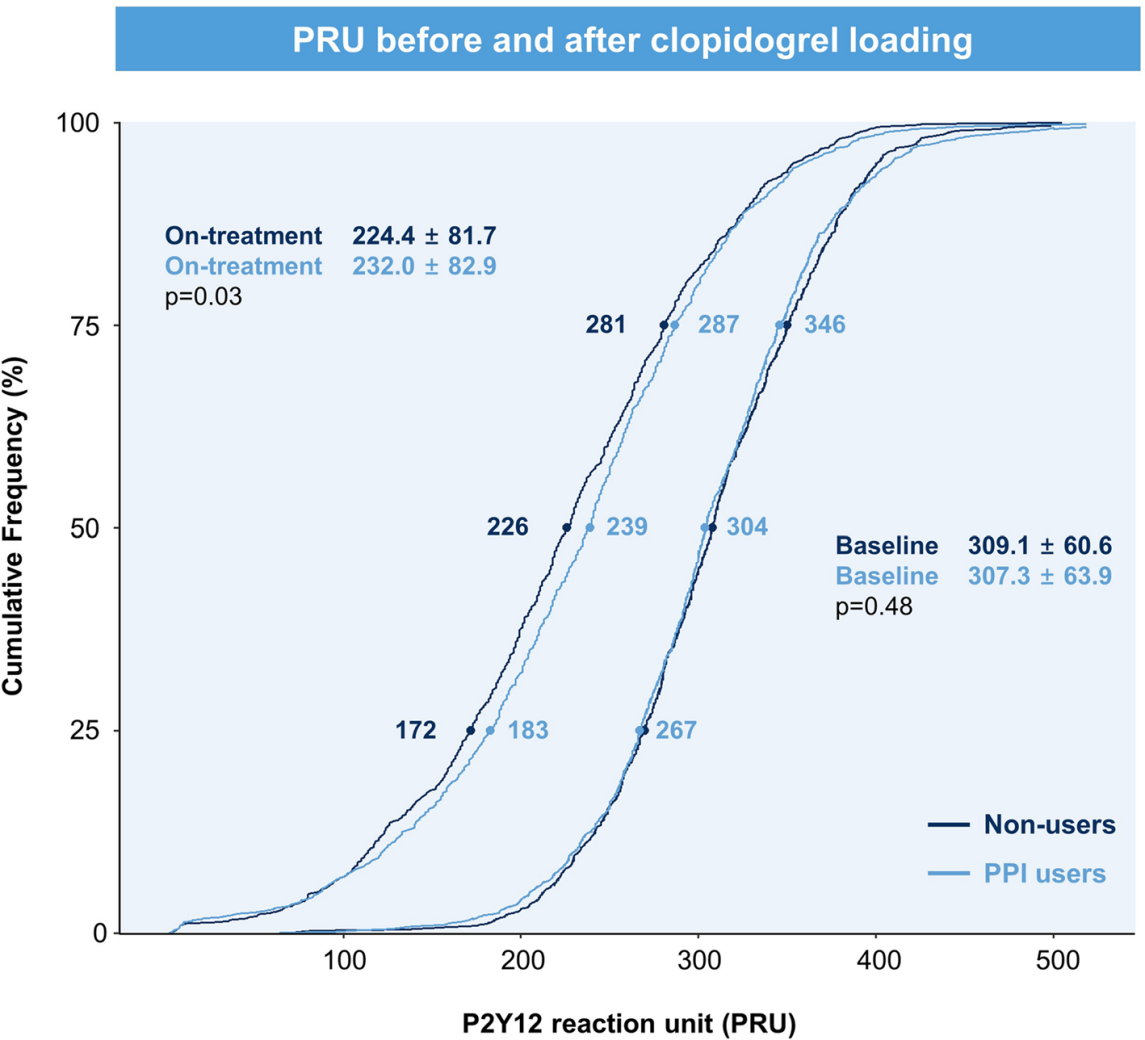
Distribution of the PRU before and after clopidogrel loading in the PS-matched cohort. The VerifyNow PRU results showed that baseline PRU values were similar between PPI users and non-users, with average values of 307.3 ± 63.9 and 309.1 ± 60.6, respectively (*p* = 0.48). However, after clopidogrel loading, on-treatment PRU values were significantly higher in PPI users (232.0 ± 82.9) than in non-users (224.4 ± 81.7). PPI, proton pump inhibitor; PRU, P2Y12 reaction unit; PS, propensity-score

Of the 13,160 patients, 2,235 (17.0%) were prescribed PPI, with an average age of 65.4 years, and 64.0% were men (Additional file 1: Table S1). PPI users were older, more frequently presented with acute MI, and had higher rates of dyslipidemia, CKD, anemia, and multivessel disease. The PS matching analysis included 6,673 patients who had both baseline PRU measurements and *CYP2C19* genotyping (PPI users, *n* = 1,133; Additional file 1: Fig. S1). After 1:1 PS matching, 2,266 patients were included in the final analysis. The demographic and clinical parameters at baseline were well balanced, with a standardized mean difference < 0.1 (Table 1). PPI users showed higher on-treatment PRU levels (after clopidogrel loading) than non-users in both the unmatched and PS-matched cohorts (Table 2 and Additional file 1: Table S2). Figure 1 presents both the baseline and on-treatment PRU values in the PS-matched cohort, as a percentile plot. PPI users had larger mean stent diameters and demonstrated procedural characteristics similar to those of non-users in the PS-matched cohort.

**Table 1:** Baseline demographics and clinical characteristics

	Propensity-score matched cohort (*n* = 2266)			
	PPI users (*n* = 1133)	Non-users (*n* = 1133)	*P* value	SMD
**Index presentation**				
Presentation as acute MI, n (%)	363 (32.0%)	367 (32.4%)	0.89	-0.0076
Stable angina	388 (34.2%)	429 (37.9%)		
Unstable angina	382 (33.7%)	337 (29.7%)		
Non-ST-segment elevation MI	188 (16.6%)	231 (20.4%)		
ST-segment elevation MI	175 (15.4%)	136 (12.0%)		
Age (years)	65.1 ± 11.2	65.6 ± 11.2	0.35	-0.0391
Male, n (%)	754 (66.5%)	745 (65.8%)	0.72	-0.0168
**Co-morbidities, n (%)**				
Hypertension	677 (59.8%)	705 (62.2%)	0.25	NA
Diabetes mellitus	382 (33.7%)	398 (35.1%)	0.51	NA
Dyslipidemia	676 (59.7%)	693 (61.2%)	0.49	-0.0306
Smoking	325 (28.7%)	320 (28.2%)	0.85	0.0098
Chronic kidney disease	266 (23.5%)	266 (23.5%)	1.00	0.0000
Anemia	373 (32.9%)	410 (36.2%)	0.11	-0.0695
Obesity (BMI ≥ 25 kg/m^2^)	488 (43.1%)	463 (40.9%)	0.31	0.0446
**Previous history, n (%)**				
History of PAD	176 (15.5%)	195 (17.2%)	0.31	-0.0463
History of CHF	83 (7.3%)	93 (8.2%)	0.48	-0.0339
Previous MI	52 (4.6%)	47 (4.1%)	0.68	0.0211
Previous PCI	111 (9.8%)	131 (11.6%)	0.20	-0.0594
Previous CABG	8 (0.7%)	6 (0.5%)	0.79	0.0211
Previous stroke	81 (7.1%)	85 (7.5%)	0.81	NA
**Lab measurements**				
LV ejection fraction, %	58.3 ± 11.5	58.6 ± 10.8	0.63	NA
Hemoglobin, g/dL	13.2 ± 2.0	13.1 ± 2.0	0.08	0.0727
Total cholesterol, mg/dL	173.5 ± 43.9	175.3 ± 45.9	0.35	NA
Triglyceride, mg/dL	142.3 ± 99.1	138.8 ± 107.1	0.43	NA
LDL-cholesterol, mg/dL	107.3 ± 39.6	105.0 ± 37.7	0.19	NA
HDL-cholesterol, mg/dL	42.6 ± 11.6	43.1 ± 11.8	0.30	NA
estimated GFR	76.7 ± 27.8	76.9 ± 27.6	0.81	-0.0102
Platelet, × 10^3^/mm^3^	236.2 ± 77.0	240.0 ± 80.5	0.26	-0.0489
VerifyNow PRU, baseline	307.3 ± 63.9	309.1 ± 60.6	0.48	-0.0289
* CYP2C19* genotyping			0.08	NA
Extensive metabolizer	407 (35.9%)	459 (40.5%)		
Intermediate metabolizer	566 (50.0%)	521 (46.0%)		
Poor metabolizer	160 (14.1%)	153 (13.5%)		
**Angiographic feature, n (%)**				
Multivessel disease	475 (41.9%)	458 (40.4%)	0.50	0.0304
Bifurcation lesion	102 (9.0%)	94 (8.3%)	0.60	0.0247
Chronic total occlusion lesion	63 (5.6%)	62 (5.5%)	1.00	0.0039
PCI at LM and/or LAD	741 (65.4%)	733 (64.7%)	0.76	0.0148
**Concomitant discharge medications, n (%)**				
Aspirin	1128 (99.6%)	1128 (99.6%)	1.00	0.0000
Beta blocker	738 (65.1%)	727 (64.2%)	0.66	NA
Angiotensin blockade	770 (68.0%)	770 (68.0%)	1.00	0.0000
Calcium channel blocker	438 (38.7%)	438 (38.7%)	1.00	0.0000
Statin	1060 (93.6%)	1061 (93.6%)	1.00	-0.0036
**Discontinuation of DAPT within 1 year**	248 (21.9%)	247 (21.8%)	1.00	0.0021
Aspirin monotherapy	173	166		
Clopidogrel monotherapy	73	81		
Others	2	0		

Values are presented as numbers (percentages) or means ± standard deviation

*BMI* Body mass index, *CABG* Coronary artery bypass graft surgery, *CHF* Congestive heart failure, *DAPT* Dual antiplatelet therapy, *GFR* Glomerular filtration rate, *HbA1c* Hemoglobin A1c, *HDL* High-density lipoprotein, *LDL* Low-density lipoprotein, *LAD* Left anterior descending artery, *LM* Left main, *LV* Left ventricular, *MI* Myocardial infarction, *PAD* Peripheral artery disease, *NA* Not applicable due to exclusion from propensity-score matching, *PCI* Percutaneous coronary intervention, *PRU* P2Y12 reaction unit, *PPI* Proton pump inhibitor, *SMD* Standardized mean difference

**Table 2:** Platelet reactivity and procedural characteristics

	Propensity-score matched cohort (*n* = 2266)			
	PPI users (*n* = 1133)	Non-users (*n* = 1133)	*P* value	SMD
**Platelet reactivity**				
On-treatment PRU	232.0 ± 82.9	224.4 ± 81.7	0.03	NA
> 208	723 (63.8%)	670 (59.1%)	0.03	NA
≥ 230	614 (54.2%)	548 (48.4%)	0.01	NA
≥ 252	475 (41.9%)	435 (38.4%)	0.10	NA
**Procedural data, n (%)**				
Multivessel PCI	482 (42.5%)	458 (40.4%)	0.33	NA
Treated lesions				
Left main coronary artery	67 (5.9%)	70 (6.2%)	0.86	NA
Left anterior descending artery	703 (62.0%)	694 (61.3%)	0.73	NA
Left circumflex artery	339 (29.9%)	324 (28.6%)	0.52	NA
Right coronary artery	464 (41.0%)	433 (38.2%)	0.20	NA
Number of stents per patient, n	1.64 ± 0.8	1.62 ± 0.8	0.47	NA
Total stent length per patient, mm	37.8 ± 23.7	36.2 ± 21.5	0.09	NA
Mean stent diameter per patient, mm	3.06 ± 0.5	2.97 ± 0.4	< 0.001	NA
DES type			0.01	NA
First-generation DES	34 (3.0%)	59 (5.2%)		
Newer-generation DES	1099 (97.0%)	1074 (94.8%)		

Values are presented as numbers (percentages) or means ± standard deviation

*DES* Drug-eluting stent, *NA* Not applicable due to exclusion from propensity-score matching, *PCI* Percutaneous coronary intervention, *PRU* P2Y12 reaction unit, *PPI* Proton pump inhibitor, *SMD* Standardized mean difference

### Effect of PPIs on ischemic and bleeding events

**Fig. 2:**
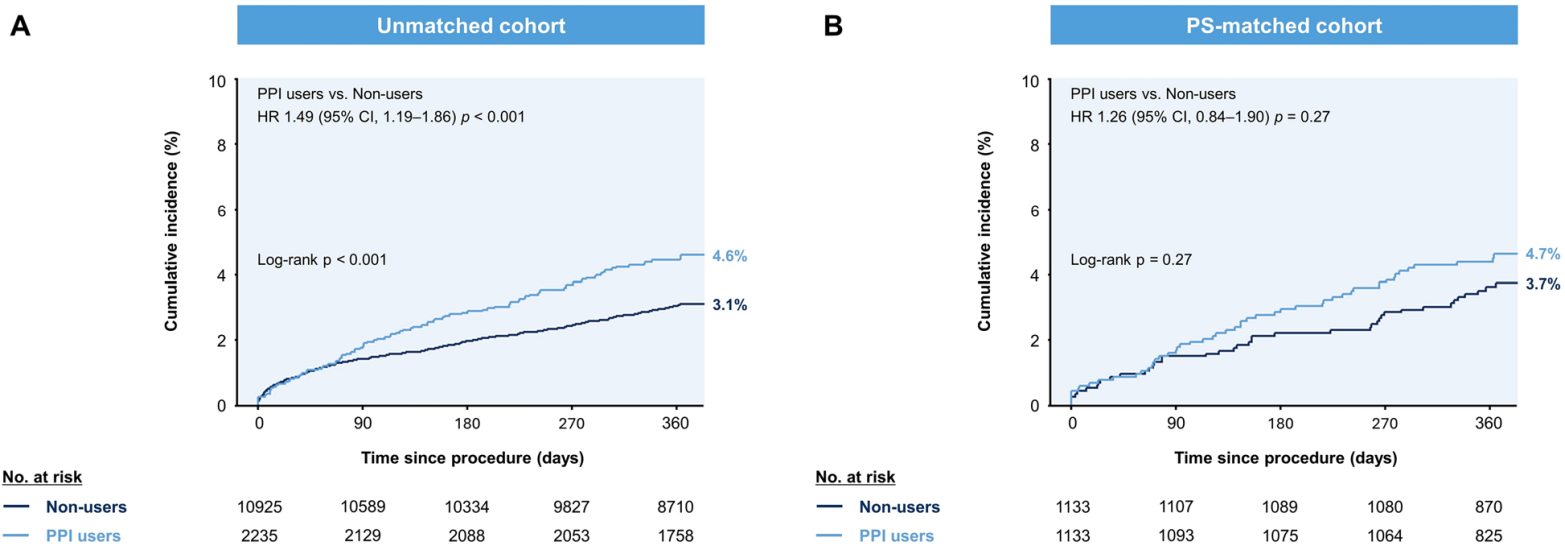
Cumulative incidence of major adverse cardiac and cerebrovascular events before and after PS matching. Before matching, the 12-month cumulative incidence of the primary outcome was higher in PPI users than in non-users (**A**). After 1:1 matching, the incidence rate was similar between PPI users and non-users (**B**). CI, confidence interval; HR, hazard ratio; PPI, proton pump inhibitor; PS, propensity-score

**Fig. 3:**
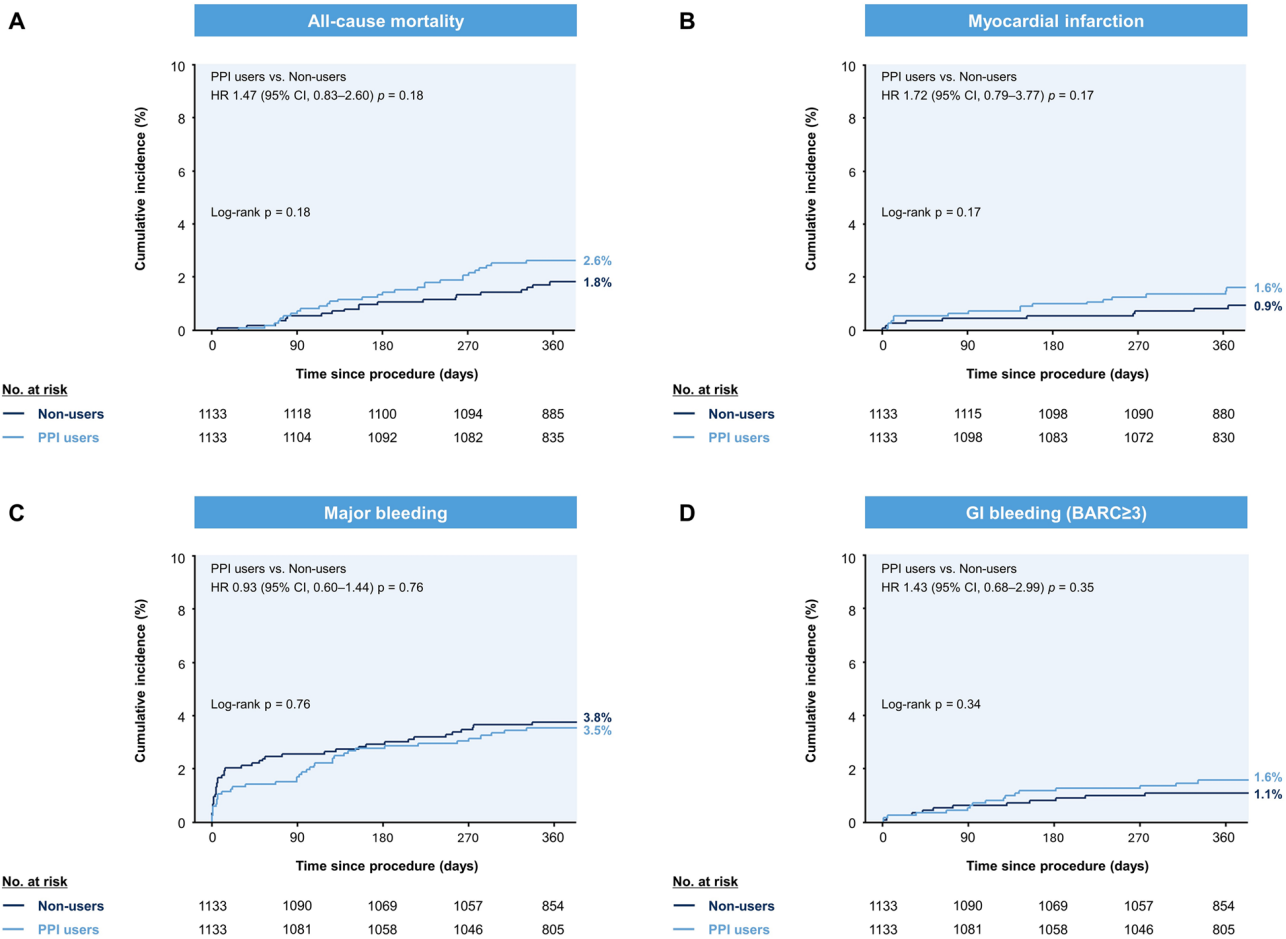
Cumulative incidence of secondary outcomes in the PS-matched cohort. Key secondary outcomes included all-cause death, with PPI users experiencing a 2.6% incidence rate versus 1.8% in non-users (**A**). PPI users showed a numerically higher incidence of spontaneous myocardial infarction than non-users (**B**). Major bleeding events were similar between groups, 3.5% for PPI users and 3.8% for non-users (**C**). Major GI bleeding also showed no significant differences between the two groups (**D**). BARC, Bleeding Academic Research Consortium; CI, confidence interval; HR, hazard ratio; GI, gastrointestinal; PPI, proton pump inhibitor; PS, propensity-score

Table 3 and Additional file 1: Table S3 summarizes the primary and secondary outcomes at 12 months according to PPI use. Before PS matching, composite MACCE occurred in 99 PPI users (cumulative incidence, 4.6%) and 326 non-users (cumulative incidence, 3.1%; log-rank *p* < 0.001). After PS matching, the incidence of MACCE at 12 months was comparable between PPI users and non-users (cumulative incidence, 4.7% vs. 3.7%; log-rank *p* = 0.27; Fig. 2). Other secondary ischemic outcomes, including MI, stent thrombosis, and cerebrovascular accidents, were similar between the two groups in the PS-matched cohort (Fig. 3). PPI users showed a numerically higher incidence of spontaneous MI than non-users (1.6% vs. 0.9%, *p* = 0.17). The Fine-Gray model further supported these findings when the discontinuation of DAPT was modelled as a single competing event (Additional file 1: Table S4).

**Table 3:** Incidence of the primary and secondary outcomes at 12 months

	Propensity-score matched cohort (*n* = 2266)			
	PPI users (*n* = 1133)	Non-users (*n* = 1133)	HR [95% CI]	Log-rank *P*
**The primary outcome**				
MACCE	51 (4.7%)	41 (3.7%)	1.26 [0.84–1.90]	0.27
**Key secondary outcomes**				
All-cause death	29 (2.6%)	20 (1.8%)	1.47 [0.83–2.60]	0.18
Major bleeding	39 (3.5%)	42 (3.8%)	0.93 [0.60–1.44]	0.76
GI bleeding (≥ BARC types 3)	17 (1.6%)	12 (1.1%)	1.43 [0.68–2.99]	0.34
**Other secondary outcomes**				
Cardiovascular death	17 (1.5%)	14 (1.3%)	1.23 [0.61–2.49]	0.57
Myocardial infarction	17 (1.6%)	10 (0.9%)	1.72 [0.79–3.77]	0.17
Cerebrovascular accident	5 (0.5%)	10 (0.9%)	0.51 [0.17–1.48]	0.20
Stent thrombosis	8 (0.7%)	6 (0.5%)	1.34 [0.46–3.86]	0.59
Any revascularization	45 (4.2%)	64 (6.1%)	0.71 [0.49–1.04]	0.08

Values are presented as numbers (an estimate of the cumulative incidence of events over time)

*BARC* Bleeding Academic Research Consortium, *CI* Confidence interval, *HR* Hazard ratio, *GI* Gastrointestinal, *MACCE* Major adverse cardiac and cerebrovascular events, *PPI* Proton pump inhibitor

In total, 257 major bleeding events occurred in 13,160 patients at 12 months, of whom 208 (80.9%) required blood transfusions. The causes of major bleeding (BARC types 3–5) included GI (42.0%, *n* = 108), central nervous system (8.6%), PCI-access site (6.6%), surgery-related (5.4%), epistaxis (3.5%), genitourinary (3.1%), pulmonary (1.9%), peripheral (1.6%), traumatic (0.8%), vascular (0.8%), pericardial (0.8%), retroperitoneal (0.4%), others (6.2%), and unknown (18.3%). Before PS matching, GI bleeding occurred in 28 PPI users (cumulative incidence, 1.3%) and 90 non-users (cumulative incidence, 0.9%; log-rank *p* = 0.05). Of these, 10 patients with GI bleeding did not meet the criteria for major bleeding. In contrast, the incidence of major bleeding and GI bleeding (BARC types 3–5) was comparable between PPI users and non-users in the PS-matched cohort. Overall, 81 major bleeding events were documented after PS matching and categorized by BARC types as follows: type 3a, 70.4%; type 3b, 18.5%; type 3c, 4.9%; type 4, 3.7%; types 5a and 5b, 1.2%. GI bleeding (BARC types 3–5) occurred in 17 PPI users (cumulative incidence, 1.6%) and 12 non-users (cumulative incidence, 1.1%; log-rank *p* = 0.34). There was only one case of non-major GI bleeding in the PS-matched cohort.

### Subgroup analysis in the PS-matched cohort

Subgroup analysis with *p* for interaction in the PS-matched cohort is shown in Table 4 (MACCE) and Additional file 1: Table S5 (major bleeding). In a subgroup analysis based on *CYP2C19* genotyping, PPI use was associated with an increased risk of MACCE (HR, 3.22;* p* = 0.02) in poor metabolizers (*n* = 313). The interaction between PPI use and *CYP2C19* genotyping was not statistically significant for MACCE (*p* for interaction = 0.28). No significant differences in MACCE were observed between PPI users and non-users in the subgroups according to hypertension, diabetes mellitus, and HPR. There were also no significant differences in major bleeding events between PPI users and non-users across various subgroups. Additional file 1: Table S6 (PS-matched cohort) and Additional file 1: Table S7 (overall cohort) describe the on-treatment PRU levels according to subgroups of genotyping and platelet reactivity.

**Table 4:** Subgroup analysis for MACCE in the propensity-score matched cohort

	No. of Patients	PPI users (*n* = 1133)	Non-users (*n* = 1133)	Log-rank *P*	Hazard Ratio (95% CI)	*P* value	*P* for interaction
***No. of MACCE events (%)***							
**Hypertension**							0.50
Yes	1382	37 (5.6%)	28 (4.1%)	0.18	1.40 [0.85–2.28]	0.18	
No	884	14 (3.3%)	13 (3.2%)	0.95	1.03 [0.48–2.18]	0.95	
**Diabetes mellitus**							0.93
Yes	780	19 (5.1%)	16 (4.1%)	0.53	1.24 [0.64–2.40]	0.53	
No	1486	32 (4.4%)	25 (3.5%)	0.35	1.28 [0.76–2.16]	0.35	
***CYP2C19* genotyping**							0.28
Extensive metabolizer	866	16 (4.1%)	13 (3.0%)	0.35	1.41 [0.68–2.94]	0.35	
Intermediate metabolizer	1087	19 (3.5%)	23 (4.5%)	0.38	0.76 [0.41–1.40]	0.38	
Poor metabolizer	313	16 (10.2%)	5 (3.3%)	0.02	3.22 [1.18–8.78]	0.02	
**Platelet reactivity**							0.60
On-treatment PRU > 208	1393	38 (5.4%)	31 (4.8%)	0.56	1.15 [0.72–1.85]	0.56	
On-treatment PRU ≤ 208	873	13 (3.3%)	10 (2.3%)	0.34	1.49 [0.65–3.39]	0.35	

Values are presented as numbers (an estimate of the cumulative incidence of events over time)

*CI* Confidence interval, *HR* Hazard ratio, *MACCE* Major adverse cardiac and cerebrovascular events, *PPI* Proton pump inhibitor, *PRU* P2Y12 reaction unit

### On-treatment PRU and clinical outcomes in the PS-matched cohort

In the PS-matched cohort, the PRU was strongly linked to the unadjusted risk of MACCE (Additional file 1: Fig. S2), and HPR was associated with a higher risk of MACCE at 12 months (Additional file 1: Fig. S3). On-treatment PRU ≥ 252 was also linked to a higher risk of MACCE at 12 months (Additional file 1: Fig. S4). The relationship between on-treatment PRU and clinical outcomes in the overall cohort has been previously reported [24].

### Predictors of 12-month MACCE in the PS-matched cohort

The independent predictors of the primary outcome were HPR and poor metabolizer status in the PS-matched cohort (Additional file 1: Table S8). Poor metabolizer was the strongest predictor of the primary outcome (adjusted HR 1.80, *p* = 0.05). In contrast, intermediate metabolizer did not present a significant risk. HPR was a significant predictor in both univariable and multivariable analyses (adjusted HR 1.73, *p* = 0.03). PPI use was not independently associated with the primary outcome (adjusted HR 1.24, *p* = 0.31).

## Discussion

This study examined the relationship among PPI usage, PRU, *CYP2C19* genotyping and adverse clinical outcomes in a large, real-world population of patients who underwent PCI with DES implantation and received clopidogrel-based DAPT. The main findings of our study were as follows: PPI use at the time of PCI was associated with higher PRU values during clopidogrel treatment, which were consistent across the *CYP2C19* genotyping subgroups; the incidence of MACCE and major bleeding events at 12 months was comparable between PPI users and non-users after PS matching; and the concomitant use of PPI and clopidogrel was associated with an increased risk of composite MACCE in poor metabolizers.

Post-discharge bleeding events are associated with increased subsequent all-cause mortality in patients with acute coronary syndromes [32]. GI bleeding significantly affects clinical outcomes in patients treated with prolonged DAPT or potent P2Y12 inhibitors after PCI [33]. PPIs are frequently co-prescribed and recommended by current guidelines to reduce the risk of GI bleeding in patients receiving DAPT or other P2Y12 inhibitors [2]. Several observational studies have suggested that coadministration of PPI can reduce the antiplatelet effects of clopidogrel [6, 9, 15, 34, 35]. However, it remains unclear whether the suggested metabolic interaction between PPI and clopidogrel leads to an increased risk of adverse ischemic outcomes, and whether PPI use is likely to be a surrogate marker of poor clinical outcomes [36, 37]. Omeprazole is the only PPI studied in RCTs, which demonstrated no apparent interaction between clopidogrel and omeprazole for cardiovascular hard endpoints [8, 38, 39]. Nevertheless, the largest RCT was prematurely terminated after a median duration of 106 days and a maximum of 341 days, and the expected prevalence of poor metabolizers within this cohort was estimated to be less than 3% [8]. To ascertain if PPIs significantly reduce clopidogrel's active metabolite levels to the extent of impairing its efficacy, a comprehensive study involving both PRU and *CYP2C19* genotyping is essential.

PPI, as a drug class, was independently associated with a PRU > 208 in patients treated with clopidogrel after successful DES implantation (PPI users; *n* = 2697) [9]. Our study uniquely describes PRU levels before and after clopidogrel loading in patients prescribed PPIs and provides results according to *CYP2C19* genotyping subgroups. The baseline PRU values were comparable in both the unmatched and PS-matched cohorts, and on-treatment PRU was significantly higher among PPI users than among non-users (232.0 ± 82.9 vs. 224.4 ± 81.7 in the PS-matched cohort). However, ischemic and bleeding events were similar between the two groups after PS matching. It is plausible that the mean difference in on-treatment PRU was too small to be clinically significant or that the interaction between PPI and clopidogrel was too weak to translate into clinical hard endpoints. Another consideration is that ischemic events appear to cluster in the higher tertile or quartile of on-treatment PRU over a certain cutoff point [40]. Although the relationship between on-treatment PRU and MACCE was largely linear in the PS-matched cohort, there was a threshold effect such that on-treatment PRU must be over 252 before a patient’s risk is elevated in the PTRG-DES cohort [23, 24]. The proportion of patients with on-treatment PRU ≥ 252 was comparable between PPI users and non-users (41.9% vs. 38.4%, *p* = 0.10) in the PS-matched cohort.

 Although European guidelines recommend the routine use of PPIs in all patients receiving DAPT, either platelet function testing or genetic testing is not recommended and may only be considered in specific situations (e.g. recurrent stent thrombosis) [2]. The PPI-clopidogrel interaction may only be clinically significant in patients with LoF *CYP2C19* alleles. To date, studies evaluating the additive effects of drug-drug and drug-gene interactions have used limited sample sizes to detect clinically meaningful differences. A recent meta-analysis found that in patients with any *CYP2C19* LoF allele, taking clopidogrel with PPIs was associated with an increased risk of adverse outcomes compared to taking clopidogrel without PPIs (*p* < 0.0001) [7]. Our study demonstrated that PPI use by poor metabolizers was associated with an increased risk of MACCE at 12 months. Meanwhile, clopidogrel-treated patients with a single LoF allele (intermediate metabolizers) can safely take PPI without a clear increase in cardiovascular risks, as shown in our data. Poor metabolizers are expected to have little or no CYP2C19 enzyme activity at baseline, and PPI use could lead to further complete inhibition of the CYP2C19 enzyme to an extent that would be clinically meaningful. This is particularly important if patients at high risk of bleeding are being considered for a de-escalation strategy with clopidogrel monotherapy, as it may inadvertently increase the risk of thrombotic events following early discontinuation of DAPT [41]. Therefore, caution should be exercised when prescribing PPI and clopidogrel without *CYP2C19* genotyping, particularly when clopidogrel monotherapy is planned.

### Limitations

Our study had few limitations. First, selection bias and unmeasured confounding factors could not be excluded despite the well-balanced PS matching results. Before PS matching, only 17% of the patients received PPI treatment, and high-risk baseline features of bleeding, such as CKD, were more frequent in PPI users. Despite performing a thorough PS matching analysis including 26 variables to adequately address any potential bias, there was a numerically higher incidence of major GI bleeding in PPI users (1.6%) than in non-users (1.1%) in the PS-matched cohort. Given the well-established benefits of PPIs for GI protection [42], those who were already susceptible to bleeding complications were more likely to receive PPIs in our cohort. This is because within the Korean reimbursement system, the prescription of PPI during DAPT is limited to patients who have other valid medical justifications, such as gastroesophageal reflux disease. The overall findings should be interpreted with caution without randomization of PPI use. However, the decision to use PPI in previous studies was also based on the clinical judgment of the physician rather than on random assignment [9, 36, 43, 44]. Second, individual PPIs were not specified, and indications for PPIs—such as dosage, duration, interruption, and termination of PPIs—were not assessed despite long-term follow-up. Most previous studies regarding this drug-drug interaction did not specify individual PPIs [12], and studies that did specify PPIs included a limited number of patients to evaluate long-term adverse outcomes [15, 45–47]. In observational analyses of prospective trials [36, 43, 44], the use of PPIs has also been evaluated as a drug class. In addition, drug exposure is an important time-dependent covariate that may affect clopidogrel-mediated platelet inhibition. In our study, the therapeutic crossover in both PPI users and non-users could have biased the results toward the null hypothesis. Third, the PTRG-DES registry covers a substantial period of inclusion from 2003 to 2018, which encompasses significant changes in clinical practice, including the evolution of drug therapy, DES technology, and PCI techniques. Finally, our analysis was restricted to the Korean population, limiting its relevance to other ethnicities with varied *CYP2C19* genotype prevalences. In the PS-matched cohort, 61.8% had any *CYP2C19* LoF allele (intermediate metabolizers, 48.0%; poor metabolizers, 13.8%), consistent with the higher frequency in East Asian patients [48, 49]. In a recent prospective trial, 23% of the patients were East Asians, and among them, 59.7% had *CYP2C19* LoF alleles [50]. East Asian patients have a higher rate of bleeding complications than white patients with similar platelet reactivity [19]. In our PS-matched cohort, major bleeding occurred in 81 patients (cumulative incidence, 3.6%) at 12 months, which is comparable to that reported in previous studies [51, 52].

## Conclusions

Despite a small but significant increase in on-treatment PRU values, the concomitant use of PPIs, when clinically indicated, in patients receiving clopidogrel-based DAPT was not associated with an increased risk of adverse cardiac and cerebrovascular events at 12 months. However, poor metabolizers who received PPIs had a higher risk of composite ischemic outcomes than that of non-users. Future research using a more individualized approach is needed to elucidate these interactions and assess the impact of alternative antiplatelet strategies in high-risk patients.

### Supplementary Information

Additional file 1: Table S1. Baseline demographics and clinical characteristics in the unmatched cohort. Table S2. Platelet reactivity and procedural characteristics in the unmatched cohort. Table S3. Incidence of clinical outcomes at 12 months in the unmatched cohort. Table S4. Multivariable adjusted model in the propensity-score matched cohort. Table S5. Subgroup analysis for major bleeding in the propensity score-matched cohort. Table S6. On-treatment PRU levels in the propensity score-matched cohort. Table S7. On-treatment PRU levels in the unmatched cohort. Table S8. Risk of the primary outcome in the propensity-score matched cohort. Fig. S1. Flow chart of the study population. Fig. S2. Spline curve for the association of the PRU as a continuous variable with the unadjusted risk of 1-year major adverse cardiac and cerebrovascular events. Fig. S3. Cumulative incidence of the primary outcome according to PRUat 12 months. Fig. S4. Cumulative incidence of the primary outcome according to PRUat 12 months.

## Data Availability

No datasets were generated or analysed during the current study.

## Abbreviations

BARC: Bleeding Academic Research Consortium
CKD: Chronic kidney disease
DAPT: Dual antiplatelet therapy
DES: Drug-eluting stent
GI: Gastrointestinal
HPR: High platelet reactivity
HR: Hazard ratio
LoF: Loss of function
MACCE: Major adverse cardiac and cerebrovascular events
MI: Myocardial infarction
PCI: Percutaneous coronary intervention
PPI: Proton pump inhibitor
PRU: P2Y12 reaction unit
PS: Propensity score
RCT: Randomized clinical trial
