# Supplementary material for: Impact of proton pump inhibitor use on clinical outcomes in East Asian patients receiving clopidogrel following drug-eluting stent implantation
Source: BMC Med. 2024 Aug 15;22:335. doi: 10.1186/s12916-024-03549-y (PMC11328459; doi:10.1186/s12916-024-03549-y)
Supplement: Supplementary file 1 — Additional file 1: Table S1. Baseline demographics and clinical characteristics in the unmatched cohort. Table S2. Platelet reactivity and procedural characteristics in the unmatched cohort. Table S3. Incidence of clinical outcomes at 12 months in the unmatched cohort. Table S4. Multivariable adjusted model in the propensity-score matched cohort. Table S5. Subgroup analysis for major bleeding in the propensity score-matched cohort. Table S6. On-treatment PRU levels in the propensity score-matched cohort. Table S7. On-treatment PRU levels in the unmatched cohort. Table S8. Risk of the primary outcome in the propensity-score matched cohort. Fig. S1. Flow chart of the study population. Fig. S2. Spline curve for the association of the PRU as a continuous variable with the unadjusted risk of 1-year major adverse cardiac and cerebrovascular events. Fig. S3. Cumulative incidence of the primary outcome according to PRUat 12 months. Fig. S4. Cumulative incidence of the primary outcome according to PRUat 12 months. [file 12916_2024_3549_MOESM1_ESM.docx]

**Impact of proton pump inhibitor use on clinical outcomes in East Asian patients receiving clopidogrel following drug-eluting stent implantation**

Additional file 1

This appendix was provided by the authors to supply readers with additional information regarding the current study.

1. **Supplemental Tables**

Table 1. Baseline demographics and clinical characteristics

Table 2. Platelet reactivity and procedural characteristics

Table 3. Incidence of clinical outcomes at 12 months

Table 4. Multivariable adjusted model in the propensity-score matched cohort

Table 5. Subgroup analysis for major bleeding

Table 6. On-treatment PRU levels in the propensity score-matched cohort

Table 7. On-treatment PRU levels in the unmatched cohort

Table 8. Risk of the primary outcome in the propensity-score matched cohort

1. **Supplemental Figures**

Figure 1. Flow chart of the study population

Figure 2. Spline curve for the association of the PRU as a continuous variable

Figure 3. Cumulative incidence of the primary outcome according to PRU (>208)

Figure 4. Cumulative incidence of the primary outcome according to PRU (≥252)

**Table S1. Baseline demographics and clinical characteristics in the unmatched cohort (n = 13,160)**

|  | Unmatched cohort (n=13,160) | | | |
| --- | --- | --- | --- | --- |
|  | PPI users  (n = 2235) | Non-users  (n = 10925) | *P* value |  |
| **Index presentation** |  |  |  |  |
| Acute MI, n (%) | 910 (40.7%) | 2816 (25.8%) | <0.001 |  |
| Age (years) | 65.4 ± 11.7 | 64.0 ± 10.7 | <0.001 |  |
| Male, n (%) | 1431 (64.0%) | 7417 (67.9%) | <0.001 |  |
| **Co-morbidities, n (%)** |  |  |  |  |
| Hypertension | 1296 (58.0%) | 6637 (60.8%) | 0.016 |  |
| Diabetes mellitus | 739 (33.1%) | 3880 (35.5%) | 0.029 |  |
| Dyslipidemia | 1344 (60.1%) | 6959 (63.7%) | 0.002 |  |
| Smoking | 644 (28.8%) | 2934 (26.9%) | 0.061 |  |
| Chronic kidney disease | 545 (24.4%) | 2330 (21.3%) | 0.002 |  |
| Anemia | 741 (33.2%) | 2604 (23.8%) | <0.001 |  |
| Obesity (BMI >25) | 923 (41.3%) | 4646 (42.5%) | 0.295 |  |
| **Previous history, n (%)** |  |  |  |  |
| History of PAD | 384 (17.2%) | 1166 (10.7%) | <0.001 |  |
| History of CHF | 208 (9.3%) | 864 (7.9%) | 0.031 |  |
| Previous MI | 105 (4.7%) | 866 (7.9%) | <0.001 |  |
| Previous PCI | 198 (8.9%) | 1539 (14.1%) | <0.001 |  |
| Previous CABG | 17 (0.8%) | 146 (1.3%) | 0.033 |  |
| Previous stroke | 169 (7.6%) | 752 (6.9%) | 0.271 |  |
| **Lab measurements** |  |  |  |  |
| LV ejection fraction, % | 57.9 ± 10.8 | 59.0 ± 10.5 | <0.001 |  |
| Hemoglobin, g/dL | 13.2 ± 2.0 | 13.6 ± 1.8 | <0.001 |  |
| Total cholesterol, mg/dL | 177.0 ± 45.2 | 172.9 ± 43.7 | <0.001 |  |
| Triglyceride, mg/dL | 145.7 ± 101.9 | 141.6 ± 95.4 | 0.094 |  |
| LDL-cholesterol, mg/dL | 111.9 ± 41.5 | 105.4 ± 42.8 | <0.001 |  |
| HDL-cholesterol, mg/dL | 44.3 ± 12.4 | 43.9 ± 12.6 | 0.179 |  |
| estimated GFR | 76.9 ± 28.4 | 77.4 ± 26.2 | 0.433 |  |
| Platelet, x10^3^/mm^3^ | 247.8 ± 81.0 | 242.8 ± 79.8 | 0.008 |  |
| VerifyNow PRU, baseline | 298.9 ± 62.1 | 298.4 ± 58.5 | 0.775 |  |
| *CYP2C19* genotyping |  |  | 0.126 |  |
| Extensive metabolizer | 492 (35.7%) | 2606 (38.4%) |  |  |
| Intermediate metabolizer | 692 (50.2%) | 3214 (47.4%) |  |  |
| Poor metabolizer | 195 (14.1%) | 964 (14.2%) |  |  |
| **Angiographic feature, n (%)** |  |  |  |  |
| Multivessel disease | 1053 (47.1%) | 4339 (39.7%) | <0.001 |  |
| Bifurcation lesion | 204 (9.1%) | 1304 (11.9%) | <0.001 |  |
| Chronic total occlusion lesion | 104 (4.7%) | 793 (7.3%) | <0.001 |  |
| PCI at LM and/or LAD | 1396 (62.5%) | 6780 (62.1%) | 0.740 |  |
| **Concomitant discharge medications, n (%)** |  |  |  |  |
| Aspirin | 2219 (99.3%) | 10612 (97.1%) | <0.001 |  |
| Beta blocker | 1395 (62.4%) | 6232 (57.0%) | <0.001 |  |
| Angiotensin blockade | 1595 (71.4%) | 6468 (59.2%) | <0.001 |  |
| Calcium channel blocker | 595 (26.6%) | 2523 (23.1%) | <0.001 |  |
| Statin | 2104 (94.1%) | 9503 (87.0%) | <0.001 |  |
| **Discontinuation of DAPT within 1 year** | 388 (17.4%) | 2323 (21.3%) | <0.001 |  |
| Aspirin monotherapy | 234 | 1423 |  |  |
| Clopidogrel monotherapy | 152 | 899 |  |  |
| Others | 2 | 1 |  |  |

Values are presented as numbers (percentages) or means ± standard deviation. BMI, body mass index; CABG, coronary artery bypass graft surgery; CHF, congestive heart failure; DAPT, dual antiplatelet therapy; GFR, glomerular filtration rate; HbA1c, Hemoglobin A1c; HDL, high-density lipoprotein; LDL, low-density lipoprotein; LAD, left anterior descending artery; LM, left main; LV, left ventricular; MI, myocardial infarction; PAD, peripheral artery disease; PCI, percutaneous coronary intervention; PRU, P2Y12 reaction unit; PPI, proton pump inhibitor.

**Table S2. Platelet reactivity and procedural characteristics in the unmatched cohort (n = 13,160)**

|  | Unmatched cohort (n=13,160) | | | |
| --- | --- | --- | --- | --- |
|  | PPI users  (n = 2235) | Non-users  (n = 10925) | *P* value |  |
| **Platelet reactivity** |  |  |  |  |
| On-treatment | 224.8 ± 82.2 | 216.3 ± 77.9 | <0.001 |  |
| >208 | 1197 (60.1%) | 5350 (55.0%) | <0.001 |  |
| ≥230 | 986 (49.5%) | 4309 (44.3%) | <0.001 |  |
| ≥252 | 752 (37.8%) | 3249 (33.4%) | <0.001 |  |
| **Procedural data, n (%)** |  |  |  |  |
| Multivessel PCI | 1061 (47.5%) | 4344 (39.8%) | <0.001 |  |
| Treated lesions |  |  |  |  |
| Left main coronary artery | 111 (5.0%) | 548 (5.0%) | 0.964 |  |
| Left anterior descending artery | 1333 (59.6%) | 6424 (58.8%) | 0.476 |  |
| Left circumflex artery | 642 (28.7%) | 3291 (30.1%) | 0.197 |  |
| Right coronary artery | 890 (39.8%) | 4128 (37.8%) | 0.075 |  |
| Number of stents per patient, n | 1.60 ± 0.8 | 1.56 ± 0.8 | 0.018 |  |
| Total stent length per patient, mm | 36.8 ± 22.6 | 34.8 ± 21.7 | <0.001 |  |
| Mean stent diameter per patient, mm | 3.10 ± 0.5 | 3.02 ± 0.4 | <0.001 |  |
| DES type |  |  | <0.001 |  |
| First-generation DES | 202 (9.0%) | 1732 (15.9%) |  |  |
| Newer-generation DES | 2033 (91.0%) | 9193 (84.1%) |  |  |

Values are presented as numbers (percentages) or means ± standard deviation. DES, drug-eluting stent; PCI, percutaneous coronary intervention; PRU, P2Y12 reaction unit; PPI, proton pump inhibitor.

**Table S3. Incidence of clinical outcomes at 12 months in the unmatched cohort (n = 13,160)**

|  |  | Unmatched cohort (n=13,160) | | | |
| --- | --- | --- | --- | --- | --- |
|  |  | PPI users  (n = 2235) | Non-users  (n = 10925) | HR [95% CI] | Log-rank *P* |
| **The primary outcome** |  |  |  |  |  |
| MACCE |  | 99 (4.6%) | 326 (3.1%) | 1.49 [1.19–1.86] | <0.001 |
| **Key secondary outcomes** |  |  |  |  |  |
| All-cause death |  | 55 (2.6%) | 152 (1.5%) | 1.77 [1.30–2.40] | <0.001 |
| Major bleeding |  | 54 (2.5%) | 203 (1.9%) | 1.30 [0.97–1.76] | 0.082 |
| GI bleeding (major and minor) |  | 28 (1.3%) | 90 (0.9%) | 1.53 [1.00–2.33] | 0.049 |
| GI bleeding (major) |  | 26 (1.2%) | 82 (0.8%) | 1.56 [1.00–2.42] | 0.047 |
| **Other secondary outcomes** |  |  |  |  |  |
| Cardiovascular death |  | 26 (1.2%) | 75 (0.7%) | 1.69 [1.08–2.64] | 0.019 |
| Myocardial infarction |  | 26 (1.2%) | 62 (0.6%) | 2.05 [1.30–3.24] | 0.002 |
| Cerebrovascular accident |  | 15 (0.7%) | 95 (0.9%) | 0.77 [0.45–1.33] | 0.352 |
| Stent thrombosis |  | 14 (0.6%) | 55 (0.5%) | 1.25 [0.69–2.24] | 0.462 |
| Any revascularization |  | 65 (3.1%) | 396 (3.9%) | 0.80 [0.62–1.04] | 0.099 |

Values are presented as numbers (an estimate of the cumulative incidence of events over time). CI, confidence interval; HR, hazard ratio; GI, gastrointestinal; MACCE, major adverse cardiac and cerebrovascular events; PPI, proton pump inhibitor.

**Table S4. Multivariable adjusted model in the propensity-score matched cohort (n = 2266)**

|  | Cox proportional hazards model | | | | | Fine-Gray sub-distribution hazard models | | | | |
| --- | --- | --- | --- | --- | --- | --- | --- | --- | --- | --- |
|  | No. of events | Unadjusted  HR (95% CI) | *P* value | Adjusted*  HR (95% CI) | *P* value | No. of events | Unadjusted  HR (95% CI) | *P* value | Adjusted*  HR (95% CI) | *P* value |
|  | **MACCE** |  |  |  |  | **MACCE** |  |  |  |  |
| PPI users (n=1133) | 51 | 1.26 [0.84–1.90] | 0.27 | 1.24 [0.82–1.87] | 0.31 | 49 | 1.35 [0.88–2.06] | 0.17 | 1.33 [0.87–2.04] | 0.19 |
| Non-users (n=1133) | 41 | 1.00 [Reference] |  |  |  | 37 | 1.00 [Reference] |  |  |  |
|  | **All-cause death** |  |  |  |  | **All-cause death** |  |  |  |  |
| PPI users (n=1133) | 29 | 1.47 [0.83–2.60] | 0.19 | 1.42 [0.80–2.51] | 0.23 | 27 | 1.52 [0.84–2.76] | 0.17 | 1.47 [0.81–2.68] | 0.21 |
| Non-users (n=1133) | 20 | 1.00 [Reference] |  |  |  | 18 | 1.00 [Reference] |  |  |  |
|  | **Myocardial infarction** |  |  |  |  | **Myocardial infarction** |  |  |  |  |
| PPI users (n=1133) | 17 | 1.72 [0.79–3.77] | 0.17 | 1.75 [0.80–3.82] | 0.16 | 15 | 1.91 [0.81–4.50] | 0.14 | 1.96 [0.83–4.61] | 0.13 |
| Non-users (n=1133) | 10 | 1.00 [Reference] |  |  |  | 8 | 1.00 [Reference] |  |  |  |
|  | **Stent thrombosis** |  |  |  |  | **Stent thrombosis** |  |  |  |  |
| PPI users (n=1133) | 8 | 1.34 [0.46–3.86] | 0.59 | 1.32 [0.46–3.82] | 0.61 | 8 | 1.61 [0.53–4.90] | 0.41 | 1.60 [0.55–4.72] | 0.39 |
| Non-users (n=1133) | 6 | 1.00 [Reference] |  |  |  | 5 | 1.00 [Reference] |  |  |  |
|  | **CVA** |  |  |  |  | **CVA** |  |  |  |  |
| PPI users (n=1133) | 5 | 0.51 [0.17–1.48] | 0.20 | 0.49 [0.17–1.43] | 0.19 | 5 | 0.56 [0.19–1.68] | 0.30 | 0.55 [0.18–1.63] | 0.28 |
| Non-users (n=1133) | 10 | 1.00 [Reference] |  |  |  | 9 | 1.00 [Reference] |  |  |  |
|  | **Major bleeding** |  |  |  |  | **Major bleeding** |  |  |  |  |
| PPI users (n=1133) | 39 | 0.93 [0.60–1.44] | 0.76 | 0.95 [0.62–1.47] | 0.83 | 38 | 0.91 [0.59–1.41] | 0.68 | 0.93 [0.60–1.44] | 0.73 |
| Non-users (n=1133) | 42 | 1.00 [Reference] |  |  |  | 42 | 1.00 [Reference] |  |  |  |
|  | **GI bleeding** |  |  |  |  | **GI bleeding** |  |  |  |  |
| PPI users (n=1133) | 17 | 1.43 [0.68–2.99] | 0.35 | 1.45 [0.69–3.03] | 0.33 | 16 | 1.35 [0.64–2.85] | 0.44 | 1.35 [0.64–2.85] | 0.43 |
| Non-users (n=1133) | 12 | 1.00 [Reference] |  |  |  | 12 | 1.00 [Reference] |  |  |  |

Values are presented as numbers. *Adjusted for hypertension, diabetes, previous stroke, *CYP2C19* genotyping and on-treatment P2Y12 reaction unit >208. CI, confidence interval; CVA, cerebrovascular accident; HR, hazard ratio; GI, gastrointestinal; MACCE, major adverse cardiac and cerebrovascular events; PS, propensity-score; PPI, proton pump inhibitor.

**Table S5. Subgroup analysis for major bleeding in the propensity score-matched cohort (n = 2266)**

|  | No. of  Patients | PPI users  (n = 1133) | Non-users  (n = 1133) | Log-rank *P* | Hazard Ratio  (95% CI) | *P* value | *P* for interaction |
| --- | --- | --- | --- | --- | --- | --- | --- |
| ***No. of major bleeding events (%)*** | | | | | | | |
| **Hypertension** |  |  |  |  |  |  | 0.78 |
| Yes | 1382 | 31 (4.7%) | 35 (5.0%) | 0.75 | 0.92 [0.57;1.50] | 0.75 |  |
| No | 884 | 8 (1.8%) | 7 (1.7%) | 0.87 | 1.09 [0.39;3.00] | 0.87 |  |
| **Diabetes mellitus** |  |  |  |  |  |  | 0.12 |
| Yes | 780 | 23 (6.2%) | 18 (4.6%) | 0.36 | 1.34 [0.72;2.47] | 0.36 |  |
| No | 1486 | 16 (2.2%) | 24 (3.3%) | 0.19 | 0.66 [0.35;1.24] | 0.19 |  |
| ***CYP2C19* genotyping** |  |  |  |  |  |  | 0.39 |
| Extensive metabolizer | 866 | 15 (3.8%) | 20 (4.4%) | 0.62 | 0.84 [0.43–1.65] | 0.62 |  |
| Intermediate metabolizer | 1087 | 17 (3.1%) | 18 (3.5%) | 0.69 | 0.87 [0.45–1.70] | 0.69 |  |
| Poor metabolizer | 313 | 7 (4.5%) | 4 (2.8%) | 0.38 | 1.73 [0.51–5.92] | 0.38 |  |
| **Platelet reactivity** |  |  |  |  |  |  | 0.17 |
| On-treatment PRU >208 | 1393 | 25 (3.5%) | 31 (4.7%) | 0.28 | 0.75 [0.44–1.27] | 0.28 |  |
| On-treatment PRU ≤208 | 873 | 14 (3.5%) | 11 (2.4%) | 0.36 | 1.45 [0.66–3.19] | 0.36 |  |

Values are presented as numbers (an estimate of the cumulative incidence of events over time). CI, confidence interval; HR, hazard ratio; PPI, proton pump inhibitor; PRU, P2Y12 reaction unit.

**Table S6.** **On-treatment PRU levels in the propensity score-matched cohort (n = 2266)**

|  | No. of  Patients | PPI users  (n = 1133) | Non-users  (n = 1133) | *P* value |
| --- | --- | --- | --- | --- |
| ***CYP2C19* genotyping** |  |  |  |  |
| Extensive metabolizer | 866 | (n = 407) | (n = 459) |  |
|  |  | 208.6 ± 82.5 | 201.8 ± 82.3 | 0.23 |
| Intermediate metabolizer | 1087 | (n = 566) | (n = 521) |  |
|  |  | 239.6 ± 80.5 | 235.0 ± 76.6 | 0.34 |
| Poor metabolizer | 313 | (n = 160) | (n = 153) |  |
|  |  | 264.6 ± 76.5 | 256.2 ± 79.0 | 0.34 |
| **Platelet reactivity** |  |  |  |  |
| On-treatment PRU >208 | 1393 | (n = 723) | (n = 670) |  |
|  |  | 280.8 ± 51.0 | 278.5 ± 49.5 | 0.41 |
| On-treatment PRU ≤208 | 873 | (n = 410) | (n = 463) |  |
|  |  | 145.7 ± 52.5 | 146.1 ± 49.0 | 0.90 |

Values are presented as means ± standard deviation. PPI, proton pump inhibitor; PRU, P2Y12 reaction unit.

**Table S7. On-treatment PRU levels in the unmatched cohort (n = 13,160)**

|  | No. of  Patients | PPI users  (n = 2235) | Non-users  (n = 10925) | *P* value |
| --- | --- | --- | --- | --- |
| ***CYP2C19* genotyping** |  |  |  |  |
| Extensive metabolizer | 3098 | (n = 492) | (n = 2606) |  |
|  |  | 208.7 ± 82.4 | 191.9 ± 78.7 | <0.001 |
| Intermediate metabolizer | 3906 | (n = 692) | (n = 3214) |  |
|  |  | 239.2 ± 80.9 | 221.9 ± 71.1 | <0.001 |
| Poor metabolizer | 1159 | (n = 195) | (n = 964) |  |
|  |  | 264.6 ± 76.5 | 249.7 ± 74.3 | 0.022 |
| **Platelet reactivity** |  |  |  |  |
| On-treatment PRU >208 | 6547 | (n = 1197) | (n = 5350) |  |
|  |  | 277.4 ± 50.3 | 272.4 ± 46.3 | 0.002 |
| On-treatment PRU ≤208 | 5167 | (n = 794) | (n = 4373) |  |
|  |  | 145.6 ± 51.9 | 147.7 ± 48.2 | 0.285 |

Values are presented as means ± standard deviation. PPI, proton pump inhibitor; PRU, P2Y12 reaction unit.

**Table S8. Risk of the primary outcome (12-month MACCE) in the propensity-score matched cohort (n = 2266)**

| **Variables** | **Univariable**  **HR (95% CI)** | ***P* value** | **Multivariable**  **HR (95% CI)** | ***P* value** |
| --- | --- | --- | --- | --- |
| Hypertension | 1.56 [1.00–2.44] | 0.05 | 1.47 [0.93–2.32] | 0.10 |
| Diabetes mellitus | 1.18 [0.77–1.80] | 0.44 | 1.07 [0.70–1.64] | 0.76 |
| Previous stroke | 1.76 [0.94–3.31] | 0.08 | 1.65 [0.87–3.11] | 0.12 |
| On-treatment PRU >208 | 1.92 [1.20–3.07] | 0.01 | 1.73 [1.07–2.80] | 0.03 |
| Intermediate metabolizer (vs. normal metabolizer) | 1.16 [0.72–1.87] | 0.53 | 1.04 [0.65–1.69] | 0.86 |
| Poor metabolizer (vs. normal metabolizer) | 2.06 [1.18–3.62] | 0.01 | 1.80 [1.01–3.18] | 0.05 |
| Proton pump inhibitor | 1.26 [0.84–1.90] | 0.27 | 1.24 [0.82–1.87] | 0.31 |

CI, confidence interval; HR, hazard ratio; MACCE, major adverse cardiac and cerebrovascular event; PRU, P2Y12 reaction unit.

**Figure S1. Flow chart of the study population**


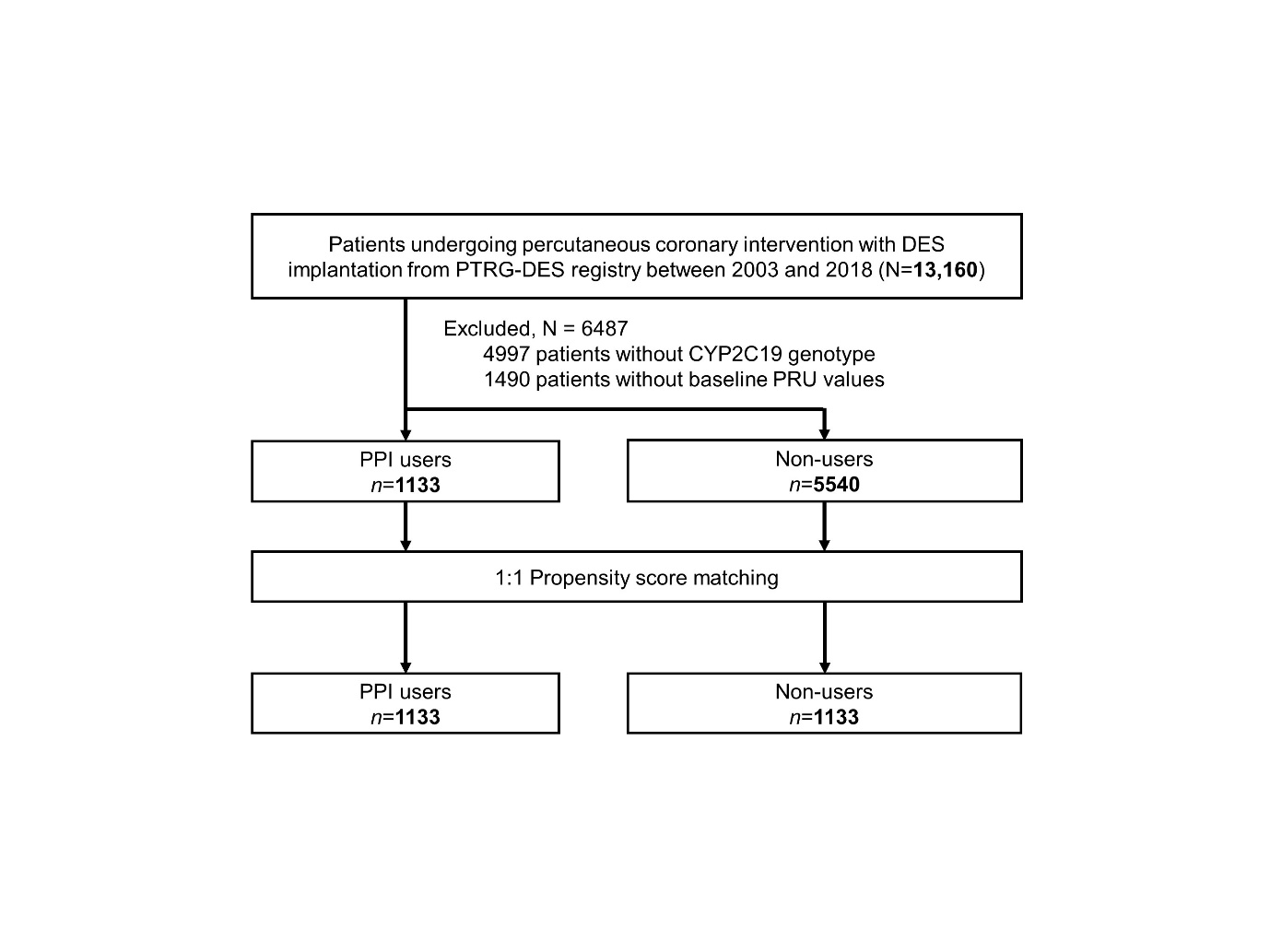


DES, drug-eluting stent; PRU, P2Y12 reaction unit; PPI, proton pump inhibitor.

**Figure S2. Spline curve for the association of the PRU as a continuous variable with the unadjusted risk of 1-year major adverse cardiac and cerebrovascular events**

**
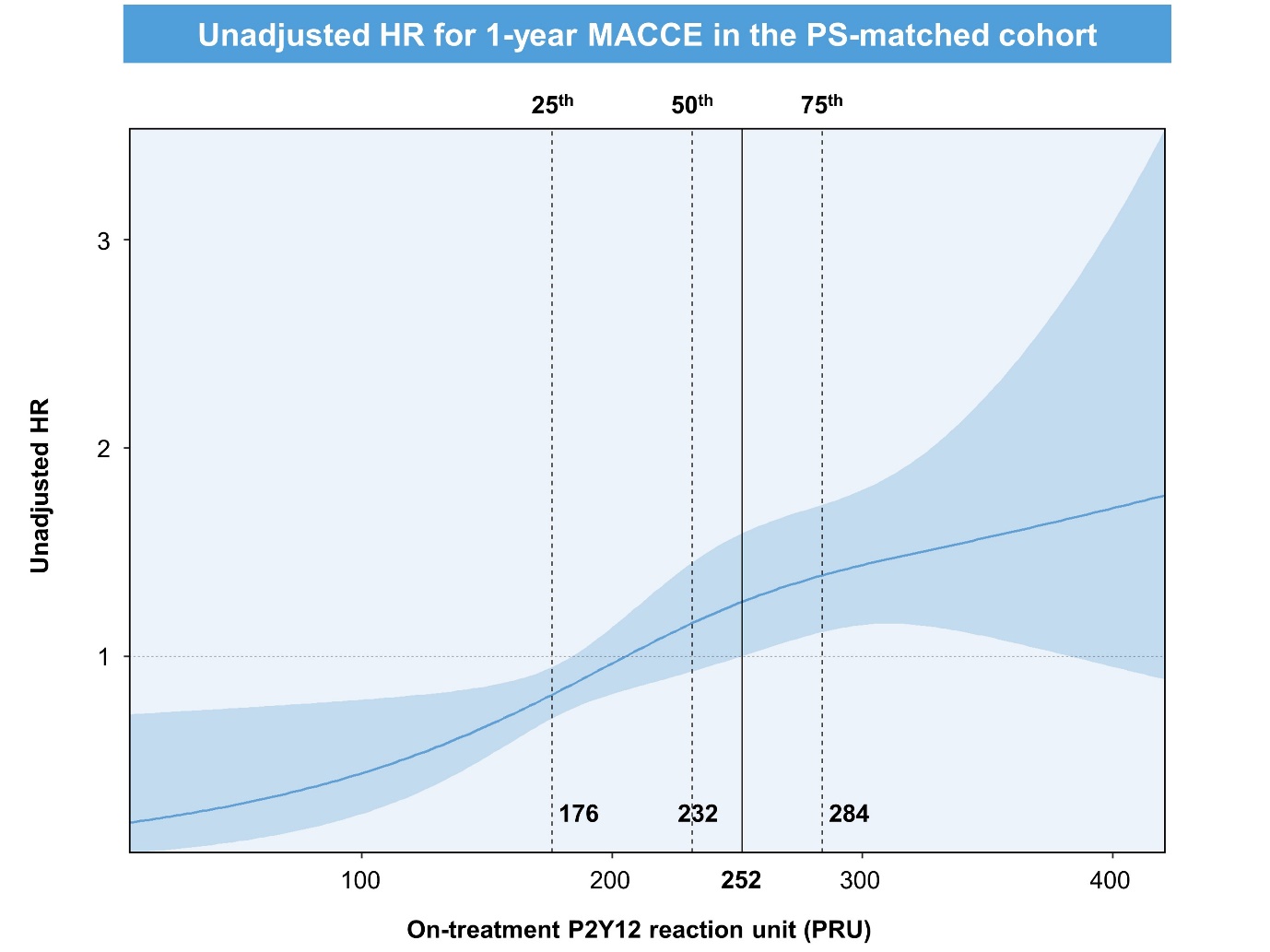
**

CI, confidence interval; HR, hazard ratio; MACCE, major adverse cardiac and cerebrovascular events; PRU, P2Y12 reaction unit; PS, propensity-score.

**Figure S3. Cumulative incidence of the primary outcome according to PRU (>208) at 12 months**

**
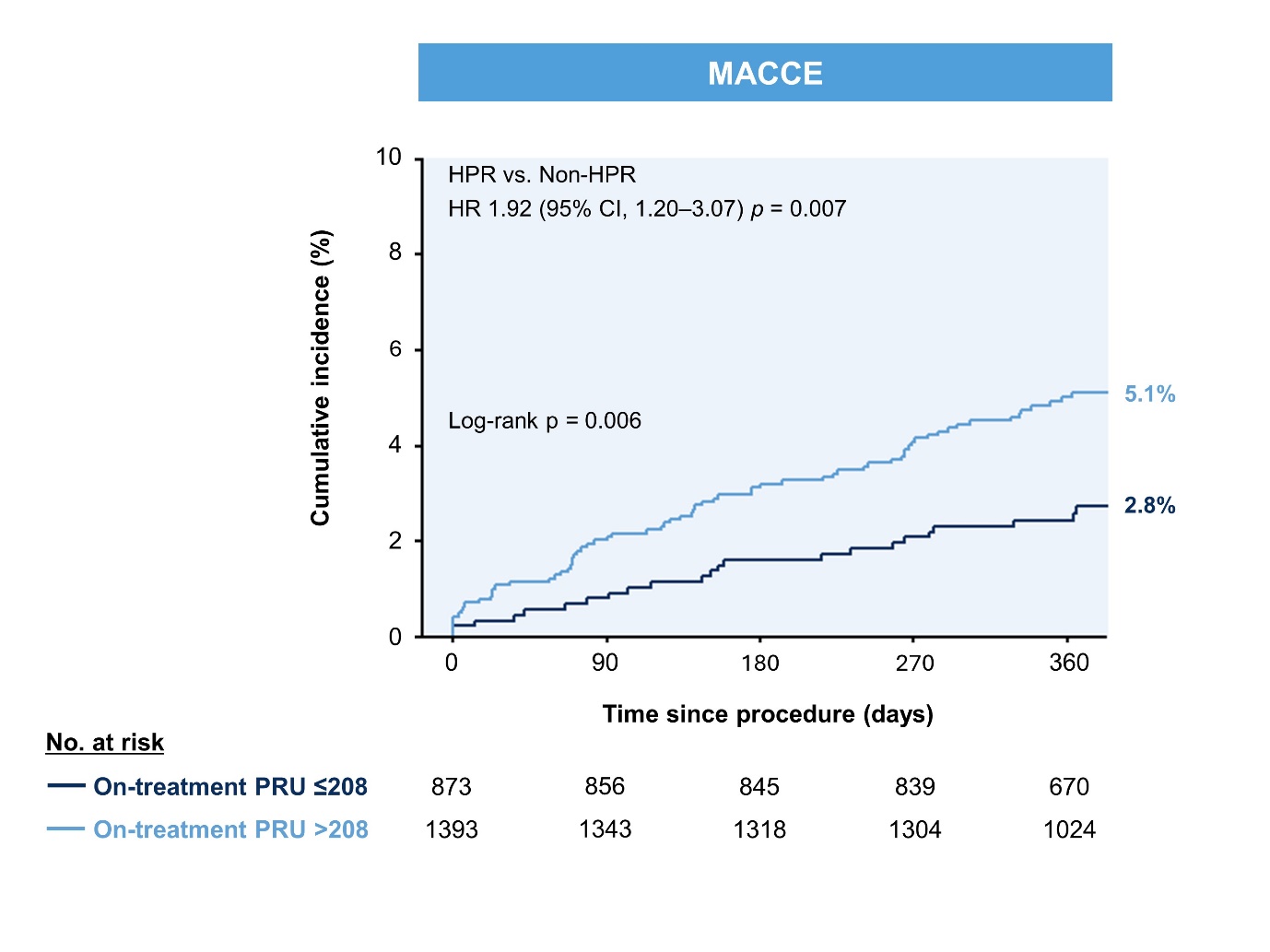
**

CI, confidence interval; HR, hazard ratio; HPR, high platelet reactivity; MACCE, major adverse cardiac and cerebrovascular events; PRU, P2Y12 reaction unit.

**Figure S4. Cumulative incidence of the primary outcome according to PRU (≥252) at 12 months**

**
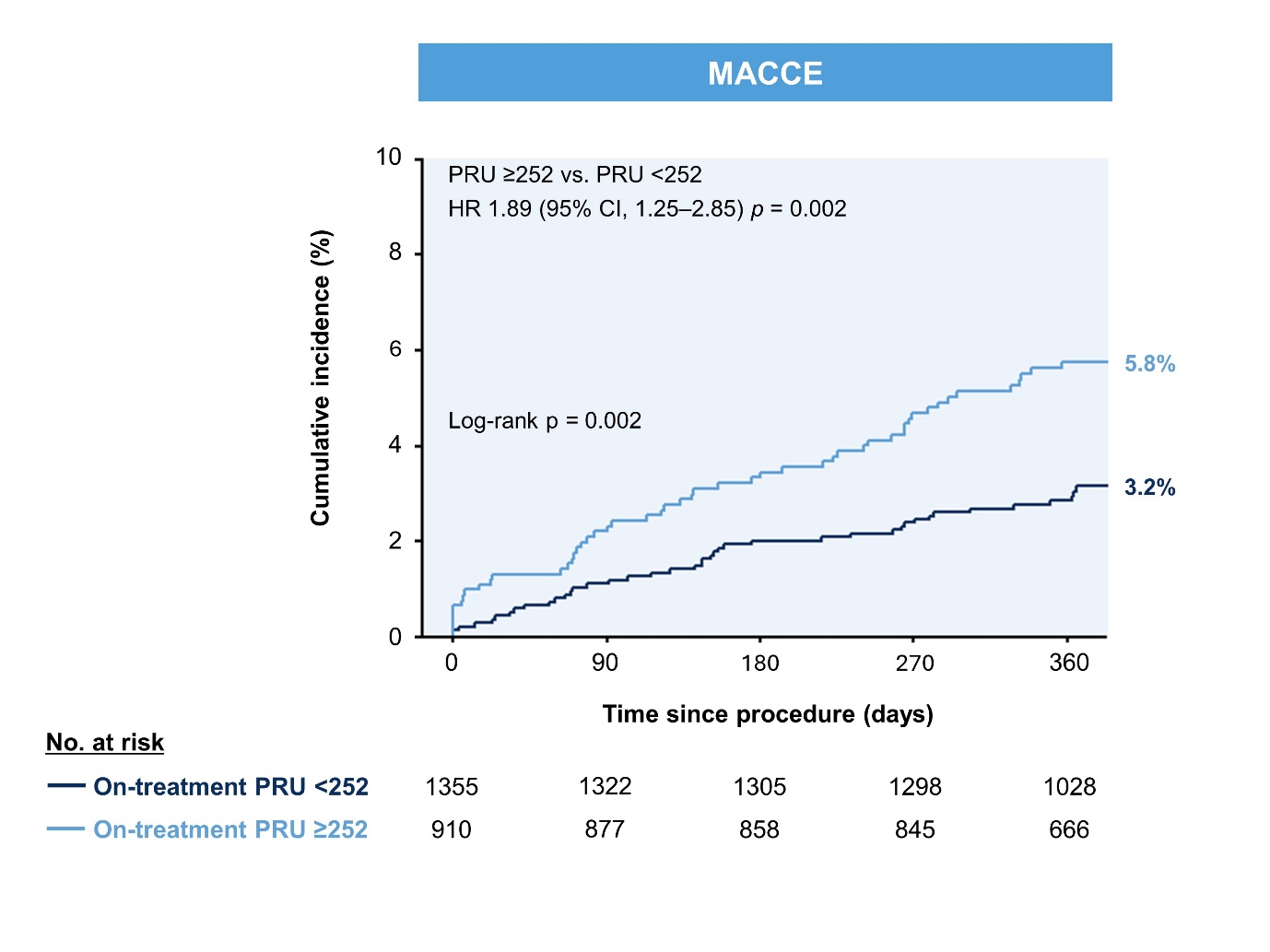
**

CI, confidence interval; HR, hazard ratio; MACCE, major adverse cardiac and cerebrovascular events; PRU, P2Y12 reaction unit.
